# Temporal Control of the Helicobacter pylori Cag Type IV Secretion System in a Mongolian Gerbil Model of Gastric Carcinogenesis

**DOI:** 10.1128/mBio.01296-20

**Published:** 2020-06-30

**Authors:** Aung Soe Lin, Mark S. McClain, Amber C. Beckett, Rhonda R. Caston, M. Lorena Harvey, Beverly R. E. A. Dixon, Anne M. Campbell, Jennifer H. B. Shuman, Neha Sawhney, Alberto G. Delgado, John T. Loh, M. Blanca Piazuelo, Holly M. Scott Algood, Timothy L. Cover

**Affiliations:** a Department of Pathology, Microbiology and Immunology, Vanderbilt University School of Medicine, Nashville, Tennessee, USA; b Department of Medicine, Vanderbilt University School of Medicine, Nashville, Tennessee, USA; c Vanderbilt Institute for Infection, Immunology, and Inflammation, Vanderbilt University Medical Center, Nashville, Tennessee, USA; d Veterans Affairs Tennessee Valley Healthcare System, Nashville, Tennessee, USA; Yale University School of Medicine

**Keywords:** *Helicobacter pylori*, type IV secretion system, gastric cancer, carcinogenesis, animal model, hit-and-run hypothesis, animal models, gene regulation

## Abstract

The Helicobacter pylori Cag type IV secretion system (T4SS) translocates the effector protein CagA and nonprotein bacterial constituents into host cells. In this study, we infected Mongolian gerbils with an H. pylori strain in which expression of the *cagUT* operon (required for Cag T4SS activity) is controlled by a TetR/*tetO* system. Transcript levels of *cagU* were significantly higher in gastric tissue from H. pylori-infected animals receiving doxycycline-containing chow (to derepress Cag T4SS activity) than in tissue from infected control animals receiving drug-free chow. At 3 months postinfection, infected animals receiving doxycycline had significantly increased gastric inflammation compared to infected control animals. Dysplasia (a premalignant histologic lesion) and/or invasive gastric adenocarcinoma were detected only in infected gerbils receiving doxycycline, not in infected control animals. We then conducted experiments in which Cag T4SS activity was derepressed during defined stages of infection. Continuous Cag T4SS activity throughout a 3-month time period resulted in higher rates of dysplasia and/or gastric cancer than observed when Cag T4SS activity was limited to early or late stages of infection. Cag T4SS activity for the initial 6 weeks of infection was sufficient for the development of gastric inflammation at the 3-month time point, with gastric cancer detected in a small proportion of animals. These experimental results, together with previous studies of *cag* mutant strains, provide strong evidence that Cag T4SS activity contributes to gastric carcinogenesis and help to define the stages of H. pylori infection during which Cag T4SS activity causes gastric alterations relevant for cancer pathogenesis.

## Abstract (precis)

The “hit-and-run model” of carcinogenesis proposes that an infectious agent triggers carcinogenesis during initial stages of infection and that the ongoing presence of the infectious agent is not required for development of cancer. H. pylori infection and actions of CagA (an effector protein designated a bacterial oncoprotein, secreted by the Cag T4SS) are proposed to constitute a paradigm for hit-and-run carcinogenesis. In this study, we report the development of methods for controlling H. pylori Cag T4SS activity *in vivo* and demonstrate that Cag T4SS activity contributes to gastric carcinogenesis. We also show that Cag T4SS activity during an early stage of infection is sufficient to initiate a cascade of cellular alterations leading to gastric inflammation and gastric cancer at later time points.

## INTRODUCTION

Helicobacter pylori has colonized the human gastric niche for at least a hundred thousand years (1–3) and is currently present in about 50% of the global population (4). H. pylori is typically acquired during childhood and commonly persists for decades or an entire lifetime (4, 5). The presence of H. pylori is a strong risk factor for gastric adenocarcinoma, and H. pylori is classified as a class I carcinogen by the World Health Organization. Gastric cancer is the third most common cause of cancer-related death worldwide (6), and H. pylori is the most common cause of noncardiac gastric cancer (7).

The risk of gastric cancer is determined in part by characteristics of the H. pylori strains with which individuals are colonized. One of the most striking genetic differences among H. pylori strains is the presence or absence of a chromosomal region known as the *cag* pathogenicity island (*cag* PAI) (8). Colonization of the human stomach with H. pylori strains containing the *cag* PAI is associated with higher gastric cancer risk than colonization with strains lacking the *cag* PAI (8). The *cag* PAI encodes CagA (a secreted bacterial oncoprotein) and a type IV secretion system (Cag T4SS) required for CagA entry into gastric cells (9, 10). Upon entry into host cells, CagA is phosphorylated by host cell kinases and interacts with multiple host cell proteins, leading to a complex array of cellular alterations that are relevant to carcinogenesis (10–12). In addition to its role in CagA secretion and entry into host cells, the Cag T4SS is required for several H. pylori-induced CagA-independent alterations in host cells, including activation of transcription factor complex NF-κB, stimulation of interleukin 8 (IL-8) production, and activation of Toll-like receptor-9. The former two phenotypes have been attributed to the intracellular entry of H. pylori lipopolysaccharide metabolites (heptose 1,7-bisphosphate or ADP heptose) (13–16), and the latter to entry of bacterial DNA into host cells (17).

Several lines of experimental evidence indicate that CagA contributes to gastric carcinogenesis. For example, experimental infection of Mongolian gerbils with a CagA-producing H. pylori strain containing an intact *cag* PAI can lead to the development of gastric cancer in infected animals, whereas infection with *cagA* mutant strains does not (18–22). Moreover, transgenic expression of CagA in mice results in tumor formation (23). In contrast to a wild-type strain containing an intact *cag* PAI, strains containing null mutations in several essential components of the Cag T4SS cause minimal gastric inflammation and do not cause gastric cancer in Mongolian gerbils (22, 24–29). Thus far, studies of Cag T4SS and CagA in animal models have compared wild-type and mutant H. pylori strains but have not included testing of complemented mutant strains.

Although human epidemiologic studies and experiments with animal models indicate that H. pylori contributes to gastric carcinogenesis, it is not known if H. pylori has carcinogenic effects mainly during early stages of infection, during later stages of infection, or throughout infection. In the early stages of gastric colonization, H. pylori proliferates in the absence of a well-developed adaptive immune response, and during this time period, the bacteria might gain access to gastric stem cell populations (30), causing mutations and other cellular alterations that initiate carcinogenesis (31–33). During later stages of infection, H. pylori resists clearance by immune defenses (34, 35) and provides a continual stimulus for gastric inflammation, which could contribute to neoplastic progression.

The hit-and-run model of carcinogenesis proposes that an infectious agent triggers carcinogenesis during initial stages of infection and that the ongoing presence of the infectious agent is not required for development of cancer (36–38). This model has been proposed for virus-induced cancers, including brain tumors (polyomavirus) (39–41), hepatocellular carcinoma (hepatitis viruses) (42), Schneiderian inverted papillomas (papillomaviruses) (43), and colorectal cancer (JC polyomaviruses) (44), and is potentially applicable to H. pylori-associated gastric cancer (12). Specifically, chronic H. pylori infection over a period of several decades can result in gastric histologic alterations (including intestinal metaplasia and atrophic gastritis) that render the stomach unsuitable for H. pylori colonization. Therefore, in some patients, H. pylori is no longer detected in the stomach at the time of gastric cancer diagnosis. It has been proposed that infection with CagA-positive H. pylori strains may act through a hit-and-run mechanism, whereby pro-oncogenic actions of CagA (translocated by the Cag T4SS) are followed by subsequent genetic or epigenetic alterations relevant to cancer pathogenesis (12). Thus far, there has been relatively little effort to experimentally test the hit-and-run model of carcinogenesis in the context of H. pylori infection, and there have not been studies to evaluate potential carcinogenic effects of CagA or Cag T4SS activity at specific time points during H. pylori infection.

In this study, we developed methodology that allowed us to conditionally regulate Cag T4SS activity in a Mongolian gerbil model of H. pylori infection. Specifically, we engineered an H. pylori strain in which the TetR/*tetO* system can be used to control expression of the *cagUT* operon, which encodes two proteins essential for Cag T4SS function (45). CagT (a VirB7 homolog) is a component of the Cag T4SS outer membrane core complex (46, 47), and CagU is predicted to be an inner membrane component of the Cag T4SS (9). We show that Cag T4SS activity contributes to development of gastric inflammation and is required for H. pylori-induced gastric carcinogenesis. In addition, experiments designed to control expression of Cag T4SS activity *in vivo* allowed us to evaluate if Cag T4SS activity contributes to gastric cancer pathogenesis during specific stages of H. pylori infection. These experiments show that derepression of Cag T4SS activity during initial stages of H. pylori infection is sufficient to initiate a cascade of cellular alterations leading to gastric inflammation at later time points when the Cag T4SS is no longer active, along with development of gastric cancer in a small proportion of animals.

## RESULTS

### Regulation of Cag T4SS activity *in vitro*.

**FIG 1:**
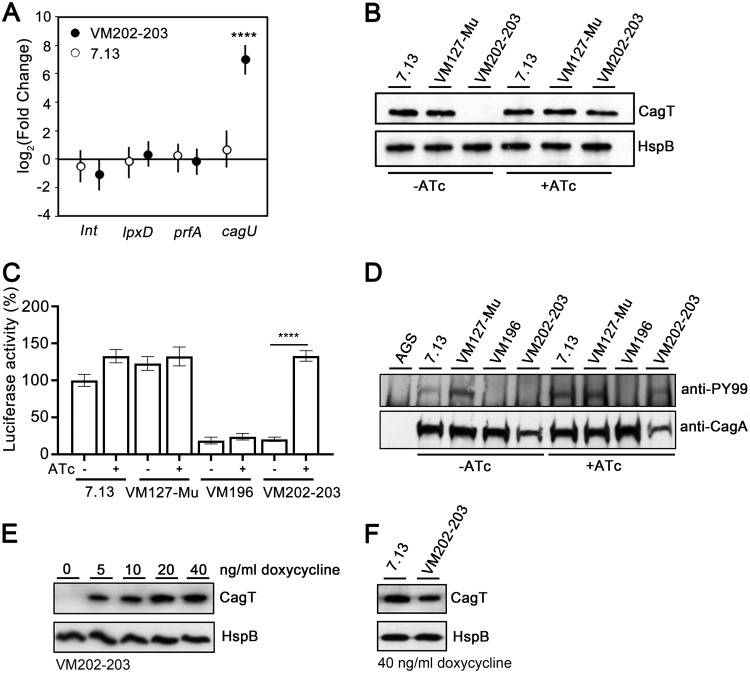
Regulatory control of *cagU* expression and Cag T4SS activity *in vitro*. (A) Transcript abundance of *cagU* and control genes (*lnt*, *lpxD*, and *prfA*) were determined as described in Text S1 in the supplemental material. Fold change values compare transcript levels of the indicated genes in H. pylori strain VM202-203 or 7.13 grown in the presence of anhydrotetracycline (ATc) to corresponding transcript levels in the same strains grown in the absence of ATc. Values represent the means and 95% credible limits (error bars). Among the genes tested, only *cagU* expression changed significantly in the presence of ATc compared with the absence of ATc. Significance was determined by calculating Bayesian z-scores, and a standard z-test was performed to derive two-tailed *P* values which were corrected for multiple testing using the Benjamini-Hochberg method (with a false discovery rate of 5%). ****, *P* ≤ 0.0001. (B) Western blot detection of CagT protein in the indicated strains in the presence (+) or absence (-) of ATc. Heat shock protein (HspB) was analyzed as a loading control. (C) NF-κB activation induced by the indicated strains in AGS reporter cells. Wild-type strains 7.13 and VM127-Mu (containing *tetR* but not *tetO*) (Table 1) were used as positive controls and VM196 (*cagU* mutant) as a negative control. The data represent results of three independent experiments with multiple technical replicates. Values represent means ± standard errors of the means (SEM). Significance was determined using the Mann-Whitney test. (D) CagA translocation into AGS gastric epithelial cells. 7.13 and VM127-Mu strains were used as positive controls and VM196 as a negative control. (E) Western blot detection of CagT in VM202-203 in the presence of subinhibitory concentrations of doxycycline (0 to 40 ng/ml). (F) Western blot detection of CagT in VM202-203 and wild-type strain 7.13 grown in the presence of 40 ng/ml doxycycline.

In a previous study, we developed a system that allowed conditional expression of Cag T4SS activity in H. pylori strain 26695, based on insertion of the *tet* repressor (*tetR*) in the *ureA* locus and insertion of *tet* operator (*tetO*) sites upstream of the *cagUT* operon (45). To facilitate experiments designed to conditionally regulate Cag T4SS in an animal model of H. pylori-induced gastric disease, we modified H. pylori strain 7.13 (a strain capable of colonizing Mongolian gerbils) so that it contained *tetR* in a locus nonessential for colonization (instead of the *ureA* locus) and *tetO* sites upstream of the *cagUT* operon, as described in Text S1 in the supplemental material (see Fig. S1 in the supplemental material). Pools of the resulting strains are designated H. pylori VM202-203 (Table 1). When strain VM202-203 was cultured in the presence of anhydrotetracycline (ATc), a derivative of tetracycline that lacks antibacterial activity, *cagU* expression was upregulated (while transcript levels of control genes remained stable), and CagT protein was produced (Fig. 1A and B). Accordingly, a Cag T4SS-dependent phenotype (NF-κB activation in AGS gastric epithelial cells) and CagA translocation into AGS cells were detected (Fig. 1C and D). When the strain was cultured in the absence of ATc, CagT protein was not produced, NF-κB activation was not detected, and CagA was not translocated into AGS gastric epithelial cells (Fig. 1B to D). Administration of ATc to Mongolian gerbils for prolonged time periods is not readily feasible due to the high cost of the drug. Therefore, we examined CagT expression in strains grown in the presence of doxycycline (another derivative of tetracycline). VM202-203 was grown in various concentrations of doxycycline. Growth was not inhibited by doxycycline concentrations up to 40 ng/ml, whereas growth was inhibited by ≥80 ng/ml (data not shown). CagT protein was produced when H. pylori was cultured in the presence of doxycycline, and the CagT levels in strains grown in 10 to 40 ng/ml doxycycline were similar to levels produced by wild-type strain 7.13 (Fig. 1E and F).

**TABLE 1:** H. pylori strains used in this study

Strain name	Description
7.13	Gerbil-adapted H. pylori strain 7.13
VM127	7.13 was transformed with plasmid pMM685, in which *tetR* and a chloramphenicol resistance determinant from pMM682 (45) were cloned into the region between *mdaB* and *hydA*.
VM127-Mu	Gerbils were infected with H. pylori VM127, and the output strain was designated VM127-Mu.
VM196	VM127-Mu in which a kanamycin resistance determinant was inserted within *cagU* (*cagU* mutant strain)
VM197-201	7.13 containing *tetR* inserted into the region between *mdaB* and *hydA* and three copies of *tetO* in proximity of the *cagUT* promoter
VM202-203	A pool of VM197-201 was used to infect gerbils. Pools of H. pylori colonies cultured from two infected gerbils were designated VM202-203.

10.1128/mBio.01296-20.1 TEXT S1 Supplemental methods. Download Text S1, DOCX file, 0.03 MB. Copyright © 2020 Lin et al. 2020 Lin et al. This content is distributed under the terms of the Creative Commons Attribution 4.0 International license.

10.1128/mBio.01296-20.2 FIG S1 Introduction of *tetR* and *tetO* in the engineered strain VM202-203. The codon-optimized *tetR* was introduced into the intergenic region between *mdaB* and *hydA* derived from strain G27. Three copies of *tetO* were introduced upstream of the *cagUT* operon to regulate *cagUT* gene expression. Download FIG S1, TIF file, 0.3 MB. Copyright © 2020 Lin et al. 2020 Lin et al. This content is distributed under the terms of the Creative Commons Attribution 4.0 International license.

### Regulation of *cagUT* expression *in vivo*.

We formulated rodent chow containing doxycycline as described in Materials and Methods. We first investigated if H. pylori VM202-203 could persistently colonize the gerbil stomach in animals receiving a diet containing doxycycline. Gerbils were experimentally infected with VM202-203, and the animals were fed chow containing a range of doxycycline concentrations (0, 50, 100, 150, or 175 mg/kg in chow) for 3 months. The animals were euthanized, and the stomachs were processed as described in Materials and Methods. In this pilot experiment, strain VM202-203 colonized the stomach in 2 out of 3 gerbils fed chow containing 50 mg/kg doxycycline and all 3 gerbils on a drug-free diet (0 mg/kg doxycycline). In contrast, H. pylori failed to colonize most of the animals fed chow containing >50 mg/kg doxycycline. The *cagU* transcript levels were higher in gastric tissue from H. pylori-infected animals receiving doxycycline than in tissue from infected control animals, based on quantitative real-time PCR (qRT-PCR) analysis (Fig. S2), indicating that expression of the *cagUT* operon could be conditionally regulated *in vivo* by doxycycline.

10.1128/mBio.01296-20.3 FIG S2 Pilot experiment analyzing transcript abundance of *cagU* and a control gene (*lpxD)* in stomach tissues of infected gerbils receiving chow containing 50 mg/kg doxycycline (*n* = 2) compared to infected gerbils receiving chow containing 0 mg/kg doxycycline (*n* = 3). Values represent the mean (and 95% credible limit) log_2_ fold change. Bayesian z-scores were calculated, and a standard z-test was performed to derive two-tailed *P* values. The *P* values (0.922 and 0.054 for *lpxD* and *cagU*, respectively) were corrected for multiple testing using the Benjamini-Hochberg method. Download FIG S2, TIF file, 0.3 MB. Copyright © 2020 Lin et al. 2020 Lin et al. This content is distributed under the terms of the Creative Commons Attribution 4.0 International license.

**FIG 2:**
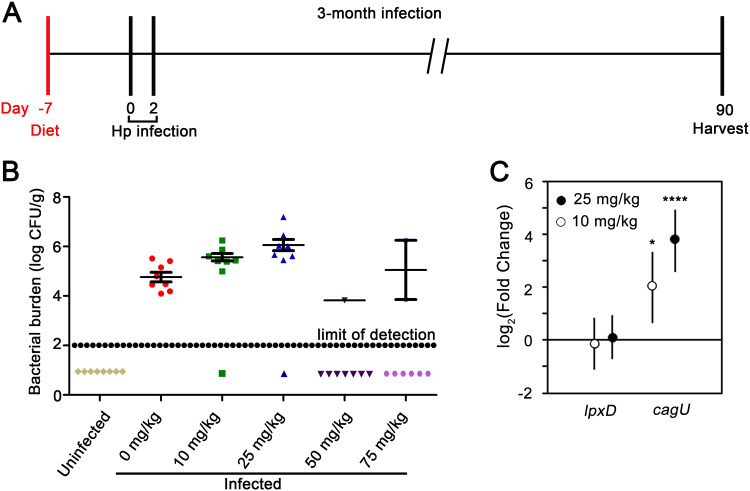
Regulatory control of *cagU* expression *in vivo*. (A) Mongolian gerbils were fed chow containing a range of doxycycline concentrations (0, 10, 25, 50, or 75 mg/kg) beginning 1 week prior to H. pylori (Hp) infection and continued for 3 months. The animals were infected with H. pylori VM202-203 via oral gavage on day 0 and day 2 and were euthanized 3 months postinfection. Uninfected animals that received drug-free chow were used as negative controls. (B) Bacterial colonization density in the stomachs of animals receiving chow containing the indicated doxycycline concentrations. The data represent values for individual animals. The data points below the limit of detection represent animals from which H. pylori could not be cultured. (C) Expression of *cagU* and a control gene (*lpxD)* in gastric tissues of infected animals fed chow containing the indicated doxycycline concentrations. Fold change values compare results for infected animals fed chow containing doxycycline compared to infected animals fed drug-free chow. Values represent the means and 95% credible limits. Transcript levels of *cagU* were significantly different in infected animals fed chow containing doxycycline (10 or 25 mg/kg) compared with animals fed a normal diet. Significance in panel C was calculated via Bayesian z-scores, and a standard z-test was performed to derive two-tailed *P* values which were corrected for multiple testing using the Benjamini-Hochberg method (with a false discovery rate of 5%). *, *P* ≤ 0.05; ****, *P* ≤ 0.0001.

To further define the optimal subantimicrobial concentration of doxycycline in gerbil chow, we infected a larger cohort of gerbils with H. pylori and fed the animals chow containing a lower range of doxycycline concentrations (0, 10, 25, 50, or 75 mg/kg in chow) for 3 months (Fig. 2A). H. pylori was successfully cultured from most of the infected animals receiving chow containing 10 or 25 mg/kg doxycycline (Fig. 2B). The H. pylori colonization density in these animals was higher than the colonization density in animals receiving drug-free chow, but this trend was not consistently detected in subsequent experiments. Thus, consumption of chow containing 10 or 25 mg/kg doxycycline did not have a substantial antimicrobial effect on H. pylori *in vivo.* In contrast, H. pylori was not successfully cultured from many of the infected animals receiving chow containing 50 or 75 mg/kg doxycycline (Fig. 2B). qRT-PCR analysis of gastric tissue indicated that consumption of chow containing 10 or 25 mg/kg doxycycline was sufficient to derepress expression of *cagU in vivo* (Fig. 2C).

### Stability of the TetR/*tetO* system *in vivo*.

We next tested if the TetR/*tetO*-dependent regulatory phenotype of H. pylori VM202-203 remained intact during colonization of the gerbil stomach for a 3-month time period (Fig. S3). We analyzed the H. pylori strains cultured from the stomachs of successfully colonized gerbils by culturing the strains in the absence or presence of ATc and testing the capacity of these strains to activate NF-κB in AGS gastric epithelial cells (as a readout of Cag T4SS activity). Seven of 8 output strains harvested from infected gerbils fed a drug-free diet induced NF-κB activation in the presence of ATc but not in the absence of ATc (Fig. S3A). Similarly, 11 of 13 output strains collected from infected gerbils fed a diet containing 10 mg/kg or 25 mg/kg doxycycline stimulated NF-κB activation in the presence of ATc but not in the absence of ATc (Fig. S3B). One strain lacking this property (4_0D [animal number four fed chow containing 0 mg/kg doxycycline]) stimulated NF-κB activation in both the presence and absence of ATc (Fig. S3A) and contained a nonsense mutation in *tetR* (data not shown). Two strains with intact *tetR/tetO* sequences were defective in NF-κB activation in both the presence and absence of ATc (2_10D and 7_25D [animal number 2 and animal number 7 fed chow containing 10 mg/kg or 25 mg/kg doxycycline, respectively]) likely contain mutations in one or more *cag* PAI genes required for T4SS function (Fig. S3B). These results indicate that the TetR-dependent regulatory properties of the Cag T4SS remained intact in H. pylori strains from most animals after colonization of the gerbil stomach for a 3-month time period. A previous study that analyzed stability of *tetR*-containing H. pylori strains in mice reached a similar conclusion (48).

10.1128/mBio.01296-20.4 FIG S3 Stability of the Cag T4SS system *in vivo*. Gerbils were infected with H. pylori VM202-203 and fed diets containing a range of doxycycline concentrations as described in the legend to Fig. 2. (A) H. pylori strains cultured from infected animals fed a normal (drug-free) diet for 3 months were tested for capacity to stimulate NF-κB activation in AGS reporter cells. The label 1-0D indicates animal number 1 fed chow containing 0 mg/kg doxycycline. (B) NF-κB activation induced by output strains cultured from infected animals fed a diet containing 10 mg/kg or 25 mg/kg doxycycline for 3 months. The labels 1-10D and 1-25D indicate strains cultured from animals fed chow containing 10 mg/kg or 25 mg/kg doxycycline, respectively. Strain 7.13 was used as a positive control and VM196 as a negative control. The individual data represent results of two or three independent experiments with multiple technical replicates. Values represent means ± standard errors of the means (SEM). Significance was determined using Mann-Whitney test for panels A and B.*, *P* ≤ 0.05; **, *P* ≤ 0.01; ***, *P* ≤ 0.001; ****, *P* ≤ 0.0001. Download FIG S3, TIF file, 0.7 MB. Copyright © 2020 Lin et al. 2020 Lin et al. This content is distributed under the terms of the Creative Commons Attribution 4.0 International license.

### Gastric inflammation in response to Cag T4SS activity.

**FIG 3:**
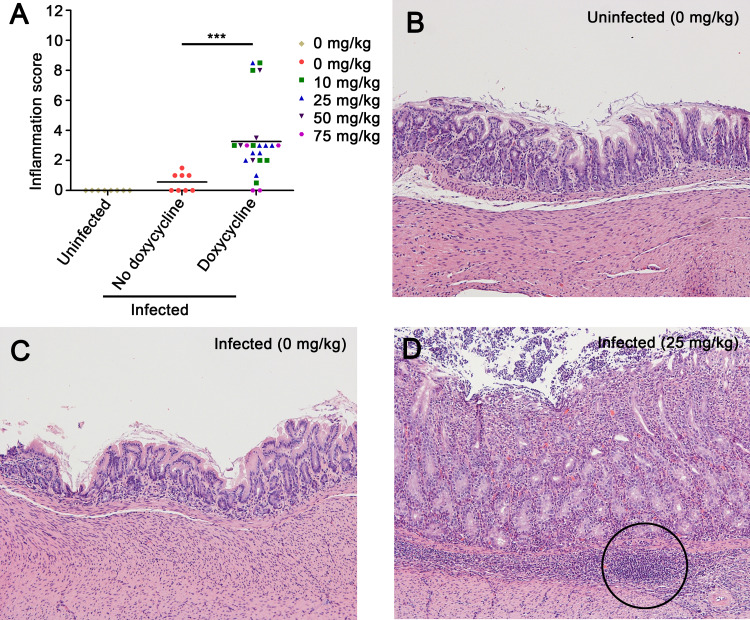
Gastric inflammation in H. pylori-infected animals in response to Cag T4SS activity. Gerbils were infected with H. pylori VM202-203 and fed diets containing a range of doxycycline concentrations as described in the legend to Fig. 2. (A) Inflammation scores in gastric mucosa. Gastric inflammation was scored on a 12-point scale as described in Materials and Methods. The data represent results for individual animals. Significance was calculated using Mann-Whitney test. ***, *P* ≤ 0.001. (B to D) Gastric antral histology from representative animals, showing normal histology in uninfected gerbils (B) and infected gerbils receiving a drug-free diet (C). (D) Severe gastric inflammation and lymphoid follicles (circle) were observed in infected gerbils receiving chow containing 25 mg/kg doxycycline. Magnification, 100×.

We next examined the gastric histology of the gerbils described above. To evaluate a potential impact of Cag T4SS activity on the severity of gastric inflammation, we used the histologic scoring system described in Materials and Methods. Gastric inflammation scores are reported for all uninfected gerbils, as well as experimentally infected gerbils from which H. pylori was successfully cultured and/or detected by use of a modified Steiner stain (Fig. 3A). Representative images of gastric histology are shown in Fig. 3B to D. As expected, gastric inflammation was not observed in tissues from uninfected animals (Fig. 3A and B). Similarly, we detected minimal gastric inflammation in H. pylori-infected animals fed a diet lacking doxycycline (Fig. 3A and C). In contrast, gastric inflammation was observed in most of the infected animals receiving chow containing 10 or 25 mg/kg doxycycline (Fig. 3A and D). The overall severity of gastric inflammation (combined analysis of antrum and corpus) in infected animals receiving doxycycline (10, 25, 50, or 75 mg/kg) was significantly greater than the severity of inflammation in infected animals receiving drug-free chow (Fig. 3A). Both acute and chronic inflammation scores (neutrophils and mononuclear cells, respectively) were significantly increased in the antrum of infected animals receiving doxycycline in chow, compared to infected animals receiving drug-free chow (Fig. S4). Dysplasia (a premalignant lesion) and/or gastric adenocarcinoma was detected in several of the H. pylori-infected animals receiving doxycycline (10, 25, or 50 mg/kg), but not in any of the infected animals receiving drug-free chow (see Table S2 in the supplemental material). These data indicate that Cag T4SS activity contributes to the development of a gastric inflammatory response and suggest that Cag T4SS activity promotes the development of gastric adenocarcinoma.

10.1128/mBio.01296-20.5 FIG S4 Gastric inflammation in antrum and corpus of infected gerbils receiving diets containing the indicated concentrations of doxycycline. Gerbils were infected with H. pylori VM202-203 and fed diets containing a range of doxycycline concentrations as described in the legend to Fig. 2. (A and B) Acute and chronic inflammation in the antrum. (C and D) Acute and chronic inflammation in the corpus. (E) Lymphoid follicles/aggregates in the glandular portion of stomach. Each symbol represents results for an individual animal. Mann Whitney test for panels A, B, C, and D or unpaired *t* test with Welch’s correction for panel E were used to calculate significance. **, *P* < 0.01; ***, *P* < 0.001. Download FIG S4, TIF file, 0.7 MB. Copyright © 2020 Lin et al. 2020 Lin et al. This content is distributed under the terms of the Creative Commons Attribution 4.0 International license.

### Temporal regulation of Cag T4SS activity.

**FIG 4:**
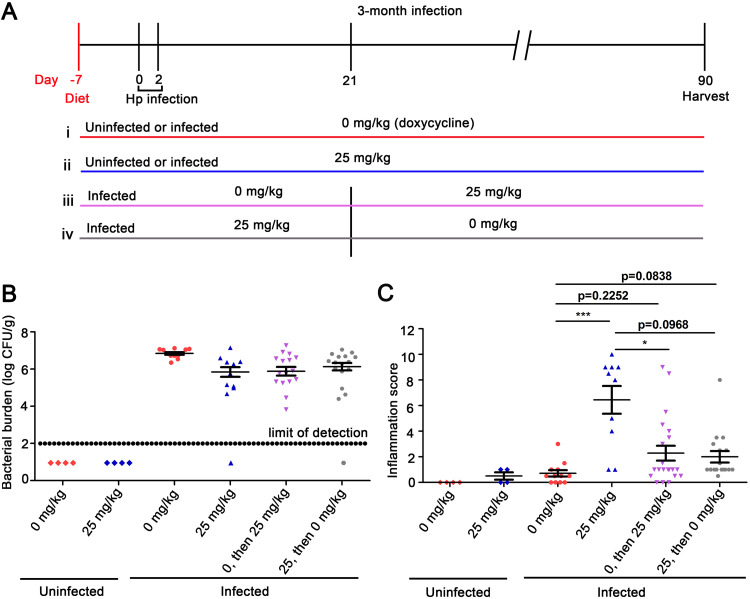
Gastric inflammation in response to Cag T4SS activity during specific stages of infection. (A) Gerbils were fed diets containing either 0 mg/kg or 25 mg/kg doxycycline 1 week prior to H. pylori infection and were infected with H. pylori VM202-203 via oral gavage on day 0 and day 2. (i and ii) H. pylori-infected gerbils or uninfected gerbils were fed diets containing either 0 mg/kg or 25 mg/kg doxycycline for the entire experiment. (iii) Gerbils were fed a diet containing 0 mg/kg doxycycline for the initial 3 weeks of infection and then switched to a diet containing 25 mg/kg doxycycline for the rest of the experiment (labeled “0, then 25” in subsequent panels). (iv) Gerbils were fed a diet containing 25 mg/kg doxycycline for the initial 3 weeks of infection and then changed to a diet containing 0 mg/kg doxycycline for the rest of the experiment (labeled “25, then 0” in subsequent panels). (B) Bacterial colonization density. (C) Inflammation scores in gastric mucosa. Significance was calculated using Kruskal-Wallis test with Dunn’s multiple-comparison test. The data represent results for individual animals. *, *P* ≤ 0.05; ***, *P* ≤ 0.001.

To further evaluate a potential role of Cag T4SS activity in gastric cancer pathogenesis, we studied larger numbers of animals and fed the animals either chow containing a standardized concentration of doxycycline (25 mg/kg) or drug-free chow. We also sought to determine if Cag T4SS activity contributed to carcinogenesis during the early stages of infection, during later stages of infection, or continuously throughout infection. Specifically, we conducted experiments in which *cagUT* expression was derepressed only during an early stage of infection (prior to development of a robust adaptive immune response and gastric inflammatory response) (49) or only during later stages of infection (Fig. 4A). One group of animals received drug-free chow for the first 3 weeks and then were switched to chow containing doxycycline (25 mg/kg) for the remaining 10 weeks of the experiment. Another group of animals received doxycycline-containing chow (25 mg/kg) for the first 3 weeks of infection and then were switched to drug-free chow for the remaining 10 weeks of the experiment. Control animals received drug-free chow or chow containing doxycycline (25 mg/kg) throughout the 13-week experiment.

Most of the animals were successfully colonized (Fig. 4B). As expected, most of the output strains from infected gerbils retained the TetR-dependent regulatory properties of the T4SS (Fig. S5). Eighteen of 20 strains induced NF-κB activation in AGS cells in the presence of ATc but not in the absence of ATc. Among the two strains lacking this property, one (8_0D [animal number eight fed chow containing 0 mg/kg doxycycline]) contained a nonsense mutation in *tetR* (data not shown), and the other (2_25D [animal number two fed chow containing 25 mg/kg doxycycline]) likely contained a mutation in a *cag* PAI gene required for T4SS activity (Fig. S5). These data confirm that H. pylori strains from most animals retained the original Cag T4SS regulatory properties after colonization of the gerbil stomach for 3 months.

10.1128/mBio.01296-20.6 FIG S5 Stability of the TetR/*tetO* system *in vivo*. Gerbils were infected with H. pylori VM202-203 and fed various diets as described in the legend to Fig. 3. (A) H. pylori strains cultured from infected animals fed a drug-free diet for 3 months were tested for their capacity to stimulate NF-κB activation in AGS cells. (B) NF-κB activation induced by output strains cultured from infected animals fed a diet containing 25 mg/kg doxycycline for 3 months. Strain 7.13 was used as a positive control and strain VM196 as a negative control. In parallel, the output strains from individual animals were grown in the absence or presence of ATc for 24 to 48 h prior to testing NF-κB activation. The data represent results of two or three independent experiments with multiple technical replicates. Values represent means ± standard errors of the means (SEM). Significance was determined using Mann-Whitney test. *, *P* ≤ 0.05; **, *P* ≤ 0.01; ***, *P* ≤ 0.001; ****, *P* ≤ 0.0001. Download FIG S5, TIF file, 0.8 MB. Copyright © 2020 Lin et al. 2020 Lin et al. This content is distributed under the terms of the Creative Commons Attribution 4.0 International license.

As expected, there was minimal gastric inflammation in uninfected animals receiving drug-free chow or chow containing 25 mg/kg doxycycline (Fig. 4C and Fig. S6 and S7). Similarly, we detected minimal inflammation in infected animals fed drug-free chow (Fig. 4C and Fig. S6 and S7). The severity of gastric inflammation in infected animals receiving doxycycline for the entire 3-month time period was significantly increased compared to the severity of inflammation in infected animals receiving drug-free chow (Fig. 4C and Fig. S6 and S7). Acute inflammation (neutrophils) and chronic inflammation (mononuclear cells) in the antrum and corpus were both significantly increased in infected animals receiving doxycycline compared to infected animals fed drug-free chow (Fig. S6). In animals receiving doxycycline for shorter time periods (only the first 3 weeks of infection or only the subsequent 10 weeks of infection), there was a trend toward increased gastric inflammation compared to infected animals receiving drug-free chow, but the differences were not statistically significant (Fig. 4C and Fig. S6 and S7).

10.1128/mBio.01296-20.7 FIG S6 Acute and chronic inflammation in response to Cag T4SS activity during specific stages of infection. Gerbils were infected with H. pylori VM202-203 and fed various diets as described in the legend to Fig. 4. (A and B) Acute and chronic inflammation in the antrum. (C and D) Acute and chronic inflammation in the corpus. (E) Lymphoid follicles/aggregates in the glandular portion of the stomach. Each symbol represents the result for an individual animal. Kruskal-Wallis test with Dunn’s multiple-comparison test was used to calculate significance for panels A, B, C and D. *, *P* < 0.05; **, *P* < 0.01; ***, *P* < 0.001. Download FIG S6, TIF file, 0.9 MB. Copyright © 2020 Lin et al. 2020 Lin et al. This content is distributed under the terms of the Creative Commons Attribution 4.0 International license.

10.1128/mBio.01296-20.8 FIG S7 Gastric inflammation in infected gerbils receiving chow containing 0 or 25 mg/kg doxycycline during defined stages of infection. Gerbils were infected with H. pylori VM202-203 and fed various diets as described in the legend to Fig. 4. (A to D) H. pylori-infected animals or uninfected animals received the indicated diets throughout the 3-month time period. (E) H. pylori-infected animals received drug-free chow during the first 3 weeks of infection, followed by chow containing doxycycline for the subsequent 10 weeks. (F) H. pylori-infected animals received chow containing doxycycline for the first 3 weeks of infection followed by drug-free chow for the subsequent 10 weeks. The panels depict gastric mucosa from the antrum, showing normal histology in uninfected gerbils receiving 0 mg/kg doxycycline (A), uninfected gerbils receiving 25 mg/kg doxycycline (B), and infected gerbils receiving 0 mg/kg doxycycline (C) and severe gastric inflammation and dysplastic glands in infected gerbils receiving 25 mg/kg doxycycline (D) and severe inflammation and lymphoid follicles in infected gerbils receiving doxycycline for the indicated time periods (E and F). Magnification, 100×. Download FIG S7, PPT file, 1.7 MB. Copyright © 2020 Lin et al. 2020 Lin et al. This content is distributed under the terms of the Creative Commons Attribution 4.0 International license.

**FIG 5:**
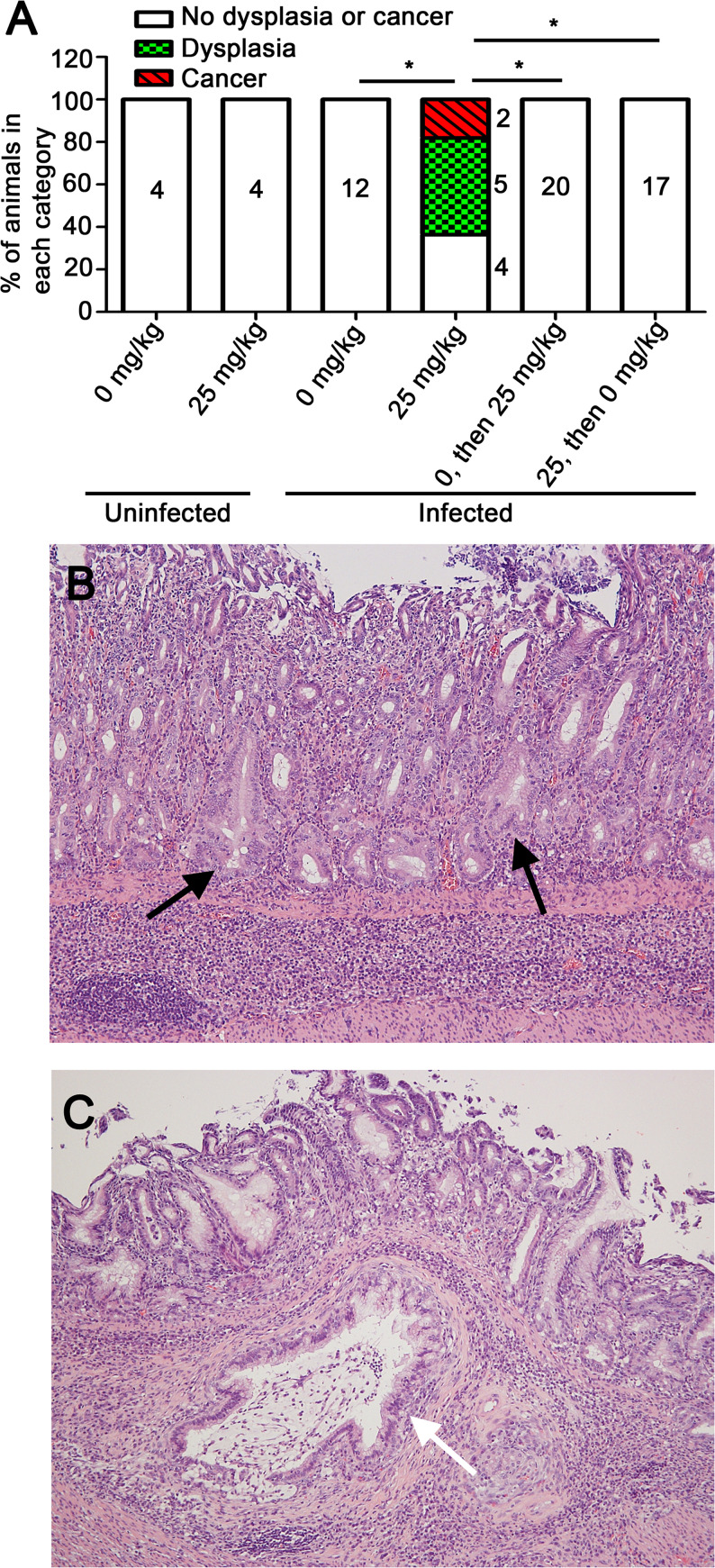
Dysplasia and gastric cancer in response to Cag T4SS activity. Gerbils were infected with H. pylori VM202-203 and fed various diets as described in the legend to Fig. 4. (A) Frequency of dysplasia and/or cancer in uninfected and infected animals receiving chow containing the indicated concentrations of doxycycline at various time points. Significance was calculated using Fisher’s exact test with Benjamini-Hochberg multitest comparison method (with a false discovery rate of 5%). *. *P* < 0.0015. (B and C) Representative gastric antral histology in infected animals, showing dysplastic glands (black arrows) and invasive carcinoma penetrating the muscularis mucosa and submucosa (white arrow). Magnification, 100×.

Dysplasia (a premalignant lesion) and/or gastric adenocarcinoma was detected in 63% of H. pylori*-*infected animals receiving doxycycline in chow for the entire 3-month time period (5/11 exhibited dysplasia, and 2/11 exhibited gastric cancer) (Fig. 5). Among 12 infected animals receiving drug-free chow, none developed dysplasia or gastric cancer (*P* = 0.0013 when comparing infected animals receiving doxycycline with animals receiving drug-free chow). Dysplasia and/or cancer were not detected in animals receiving doxycycline for less than the entire 3-month time period (drug administration for the first 3 weeks of infection or weeks 4 to 13) (Fig. 5A). These findings indicate that Cag T4SS activity is required for development of dysplasia and/or gastric cancer.

**FIG 6:**
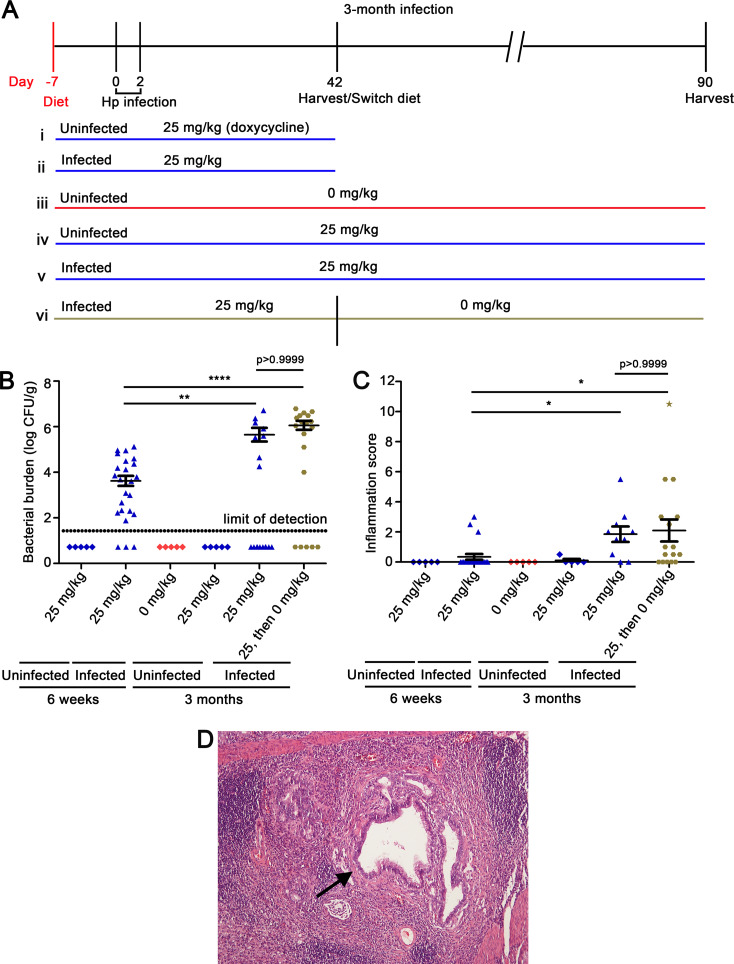
Gastric inflammation and gastric cancer in response to Cag T4SS activity during the initial 6 weeks of a 3-month infection. (A) Gerbils were fed diets containing either 0 mg/kg or 25 mg/kg doxycycline 1 week prior to infection and were infected with H. pylori VM202-203 via oral gavage on day 0 and day 2. (i and ii) H. pylori-infected or uninfected gerbils were fed diets containing 25 mg/kg doxycycline for 6 weeks. (iii and iv) Uninfected gerbils were fed diets containing either 0 mg/kg or 25 mg/kg doxycycline for the entire experiment. (v and vi) Infected gerbils were fed a diet containing 25 mg/kg doxycycline for the entire experiment or for the initial 6 weeks of infection, followed by switching to a diet containing 0 mg/kg doxycycline for weeks 7 to 13 (labeled “25, then 0” in subsequent panels). (B) Bacterial colonization density. The data represent results for individual animals. (C) Inflammation scores in gastric mucosa. The data represent results for individual animals. In this experiment, only one of the H. pylori-infected animals developed gastric adenocarcinoma (corresponding to the star-shaped data point). (D) Gastric antral histology in an infected animal (fed a doxycycline-containing diet for the initial 6 weeks of infection and then switched to a drug-free diet for the subsequent 7 weeks), showing invasive carcinoma in the submucosa. Significance was calculated using Kruskal-Wallis test with Dunn’s multiple-comparison test for panels B and C. *, *P* ≤ 0.05; **, *P* < 0.01; ****, *P* ≤ 0.0001.

Since we did not detect dysplasia or gastric cancer in animals receiving doxycycline for the first 3 weeks of a 3-month infection, we did further experiments to evaluate the duration of Cag T4SS activity during early infection that is sufficient for development of dysplasia and/or gastric cancer. Animals were fed doxycycline-containing chow for 6 weeks and sacrificed at the 6-week time point (Fig. 6A). In addition, we analyzed a group of infected animals that received doxycycline during the initial 6 weeks of infection, followed by a drug-free diet for the next 7 weeks (Fig. 6A). Control groups were similar to the groups described previously. The H. pylori colonization density was lower at the 6-week time point compared to the 13-week time point (Fig. 6B). As expected, there was minimal gastric inflammation in uninfected animals receiving either drug-free chow or chow containing doxycycline (Fig. 6C). Gastric inflammation scores were higher in infected animals euthanized at the 3-month time point (either receiving doxycycline for the first 6 weeks of infection or the entire 3-month time period) than in infected animals receiving doxycycline and euthanized at the 6-week time point (Fig. 6C). Among the infected animals in which Cag T4SS activity was derepressed for 6 weeks and then repressed for weeks 7 to 13, one developed severe gastric inflammation and gastric cancer (Fig. 6D). These results suggest that Cag T4SS activity during the initial 6 weeks of infection results in gastric inflammation at a subsequent time point when the T4SS is no longer active, and can result in gastric cancer at the later time point in a small proportion of animals.

## DISCUSSION

In this study, we utilized the TetR/*tetO* system to derepress the H. pylori *cagUT* operon, thereby allowing us to regulate Cag T4SS activity in a Mongolian gerbil model. We observed that derepression of Cag T4SS activity *in vivo* resulted in significantly higher levels of gastric inflammation than observed in infected control animals. Furthermore, dysplasia and gastric adenocarcinoma were detected only in animals in which Cag T4SS activity was derepressed. These results provide strong evidence that Cag T4SS activity contributes to development of a gastric mucosal inflammatory response and is required for development of gastric premalignant lesions and gastric cancer. Previous studies of H. pylori *cag* PAI mutant strains suggested that Cag T4SS activity contributes to gastric cancer pathogenesis in the gerbil model, but none of the previous studies tested complemented mutant strains. The approach used in the current study allowed us to overcome this limitation and also allowed us to temporally regulate Cag T4SS activity *in vivo*.

A previous study used the TetR/*tetO* system to conditionally regulate H. pylori urease in a mouse model and showed that urease is required for establishing gastric infection as well as persistence of infection (48). In the previous study, doxycycline or ATc was administered to mice in drinking water containing 5% sucrose (48). Mongolian gerbils are desert animals that drink relatively little water compared to mice. Therefore, in the current study, we fed the animals specially formulated chow containing doxycycline. Doxycycline has antibacterial activity and has been used in combination with other antibiotics for the treatment of H. pylori infection (50). In the current study, we were unable to culture H. pylori from many of the experimentally infected animals that consumed chow containing >50 mg/kg doxycycline. In contrast, administration of doxycycline in lower concentrations did not result in any detectable reduction in H. pylori colonization density compared to control gerbils receiving drug-free chow and was sufficient to derepress *cagUT* expression *in vivo*.

Although use of the TetR/*tetO* system and administration of doxycycline-containing chow allowed us to regulate Cag T4SS activity *in vivo* and detect histologic consequences of Cag T4SS activity, the incidence of premalignant changes and gastric cancer observed in the current study was somewhat lower than the corresponding incidence observed in several previous studies in which Mongolian gerbils were infected with wild-type strain 7.13 (19, 20). The lower rate of gastric cancer in the current study could potentially be due to variations among studies in study design (for example, differences in the duration of infection or differences in dietary composition). In addition, gerbils are outbred, so variations in outcomes of different studies (or variations in the severity of disease when comparing different gerbil cohorts in the current study) are potentially attributable to differences in the genetic characteristics of gerbil cohorts. Another possibility is that administration of doxycycline might modulate the severity of gastric disease that develops in response to H. pylori. For example, doxycycline administration might attenuate the severity of the gastric inflammatory response and/or lead to a reduced rate of gastric cancer.

Doxycycline can potentially have multiple activities *in vivo*, including anti-inflammatory activities (51) and effects on the intestinal microbiota (52). Doxycycline can induce esophageal ulceration mimicking a benign form of esophageal cancer (53–55), can cause gastrointestinal injury (56, 57), and can stimulate colonic tumor growth (58). On the other hand, doxycycline can also have antitumorigenic effects (59–62). In the current study, administration of doxycycline to gerbils did not cause any detected gastric histologic alterations in the absence of H. pylori infection. Therefore, the main activity of doxycycline in these experiments is attributed to its role in regulating *cagUT* expression and Cag T4SS activity.

Gastric cancer in humans is thought to arise through a multistep progression of histologic alterations (chronic gastritis, atrophic gastritis, intestinal metaplasia, dysplasia, gastric adenocarcinoma) (63). It is not known if H. pylori has carcinogenic effects mainly during early stages of infection, during later stages of infection, or throughout infection. The hit-and-run model of carcinogenesis proposes that an infectious agent triggers carcinogenesis during initial stages of infection and that the ongoing presence of the infectious agent is not required for development of cancer. This model of carcinogenesis has been implicated in multiple types of virus-induced carcinogenesis (36, 38, 64). In the current study, we experimentally tested if the hit-and-run model of carcinogenesis is applicable to Cag T4SS activity and gastric cancer, and we investigated the temporal features of Cag T4SS activity that are relevant for carcinogenesis. The results suggest that continuous Cag T4SS activity throughout a 3-month time period leads to higher rates of gastric cancer incidence than the rates observed when Cag T4SS activity is limited to early or late stages of infection. Animals in which the Cag T4SS was derepressed for a 10-week time period during later stages of infection (weeks 4 to 13) did not develop dysplasia or gastric cancer, although these animals exhibited a trend toward increased gastric inflammation compared to control infected animals receiving drug-free chow. Animals in which Cag T4SS was derepressed for the initial 3 weeks of a 3-month infection also did not develop dysplasia or cancer, but we again noted a trend toward increased gastric inflammation. Among animals in which Cag T4SS activity was derepressed for the initial 6 weeks of infection and repressed for the remainder of the 3-month experiment, one animal developed severe gastric inflammation as well as gastric cancer. The development of gastric cancer in only 1 of 19 gerbils (∼5%) in this experimental group might seem relatively low. On the other hand, fewer than 3% of H. pylori-infected humans develop gastric cancer during an entire lifetime (65). Therefore, the current results suggest that Cag T4SS activity during the initial 6 weeks of infection is sufficient to initiate cellular alterations that can result in gastric cancer in a small proportion of animals at later time points when the T4SS is no longer active, consistent with the hit-and-run model of carcinogenesis.

Cag T4SS activity probably contributes to carcinogenesis through multiple mechanisms. T4SS-mediated delivery of CagA to gastric stem cells potentially occurs throughout H. pylori infection, and likely promotes alterations in cell signaling that are procarcinogenic (30, 66). In support of this view, *in vivo* experiments indicate that H. pylori colonizes gastric glands, activates stem cells, and induces hyperplasia in mice in a CagA-dependent manner (30). During later stages of infection, Cag T4SS activity contributes to the development of a robust gastric mucosal inflammatory response (34). Enhanced gastric mucosal inflammation in the setting of persistent infection likely stimulates DNA damage through actions of reactive oxygen species and reactive nitrogen species, resulting in mutations in an assortment of genes, including p53 and genes involved in DNA repair pathways (31–33). The H. pylori Cag T4SS has also been implicated in repression of parietal cell H^+^/K^+^-ATPase expression, leading to inhibition of acid secretion during later stages of infection (67, 68). Therefore, the actions of CagA and Cag T4SS might lead to alterations in the gastric microbiome that are relevant for cancer pathogenesis (69–72). *In vitro* experiments suggest that H. pylori utilizes CagA to promote acquisition of nutrients, including iron, from the host (73). Therefore, we speculate that the Cag T4SS and CagA facilitate enhanced H. pylori replication and/or entry of H. pylori into gastric niches (for example, gastric glands adjacent to stem cells or the gastric corpus) that are less accessible in the absence of Cag T4SS activity. Further experiments will be required to decipher the precise actions of the Cag T4SS and CagA that are relevant to carcinogenesis at specific time points during infection.

The current study focused on temporal features of H. pylori Cag T4SS activity that are relevant for gastric carcinogenesis in the Mongolian gerbil model. A previous study evaluated the effect of antibiotic treatment on H. pylori-induced gastric cancer in the gerbil model (74). Specifically, Mongolian gerbils were infected with H. pylori strain 7.13 for 4 or 8 weeks, followed by treatment with antimicrobial agents for 8 weeks (74). No premalignant and malignant lesions were observed in the group of animals infected for 4 weeks and then treated with antibiotics (74). Among animals infected for 8 weeks prior to receiving antibiotics, treatment resulted in a reduced proportion of animals with premalignant or malignant lesions but did not completely prevent development of these lesions. Our finding that Cag T4SS activity for 6 weeks is sufficient for development of gastric cancer in a small proportion of animals is consistent with the results of the previous antimicrobial treatment study. In contrast to the previous study, the current study focuses specifically on the contribution of the Cag T4SS to gastric carcinogenesis.

Several studies have suggested that eradication of H. pylori with antibiotics can prevent development of gastric cancer in humans if administered prior to the development of preneoplastic gastric lesions (75). Thus, individuals with a high risk of gastric cancer can potentially benefit from antibiotic treatment if it is administered prior to the development of intestinal metaplasia and atrophic gastritis. One study suggested that antibiotic administration could prevent development of gastric cancer if administered at late stages when H. pylori is no longer present (76, 77). This effect was attributed to antibiotic-induced changes in non-H. pylori constituents of the gastric microbiome. There are several important differences between the model system used to test the hit-and-run hypothesis in this study and corresponding features in H. pylori-infected humans. H. pylori probably colonizes the human stomach for several decades prior to the development of gastric cancer. Therefore, the time period required for development of gastric cancer in H. pylori-infected humans is probably much longer than the time period required for development of gastric cancer in the gerbil model. In addition, the current study was designed to allow regulation of the Cag T4SS in animals that were continuously infected with H. pylori. In contrast, studies of the temporal relationship between H. pylori and gastric cancer in humans have been based on assessing the presence or absence of H. pylori infection.

In summary, this study demonstrates the utility of using the TetR/*tetO* system to regulate Cag T4SS activity in animal models and highlights the important role of the Cag T4SS in gastric cancer pathogenesis. Moreover, this study supports the hit-and-run model of carcinogenesis in the context of H. pylori infection, Cag T4SS activity, and development of gastric cancer. In future studies, we anticipate that use of the TetR/*tetO* system will allow investigation of the actions of additional H. pylori genes *in vivo* during different stages of infection.

## MATERIALS AND METHODS

### H. pylori culture methods.

H. pylori strains used in this study are described in Table 1. H. pylori was maintained on Trypticase soy agar (TSA) plates containing 5% sheep blood incubated at 37°C in room air supplemented with 5% CO_2_. Prior to infection of Mongolian gerbils, bacteria were inoculated into sulfite-free brucella broth supplemented with 10% fetal bovine serum (FBS) and grown to mid-log phase at 37°C in room air supplemented with 5% CO_2_. For experiments to conditionally regulate Cag T4SS activity *in vitro*, H. pylori was cultured on TSA blood agar plates with or without anhydrotetracycline (ATc; Sigma-Aldrich) (100 ng/ml) for 24 to 48 h prior to assays (*cagU* gene expression, CagT production, and NF-κB activation) (45). H. pylori was grown in broth culture for 22 h in the presence of various concentrations of doxycycline prior to analysis of CagT production. Bacteria were also cultured with or without ATc prior to testing properties of gerbil output strains (NF-κB activation).

### Generation of H. pylori strains in which Cag T4SS activity can be conditionally regulated.

In a previous study, we reported methods for conditional expression of the *cagUT* operon and Cag T4SS activity in H. pylori 26695 based on use of the TetR/*tetO* system (45). In the current study, we introduced similar TetR/*tetO* elements into the gerbil-adapted H. pylori strain 7.13 (78) as described in Text S1 in the supplemental material (see Fig. S1 in the supplemental material). The resulting strains (VM202-203 [Table 1]) were then used for experimental infection of gerbils as described below.

### Animal infection.

Male Mongolian gerbils (less than 60 g weight) were purchased from Charles River Laboratories. Gerbils were fed AIN-93M rodent diets (Bio-Serv) containing a range of doxycycline hyclate concentrations (Sigma catalog no. D9891; 0 mg/kg to 175 mg/kg in chow), beginning 1 week prior to infection with H. pylori. After fasting overnight, gerbils were infected via oral gavage (day 0 and day 2) with 1 × 10^9^ CFU of H. pylori VM202-203. To determine the optimal subantimicrobial concentration of doxycycline, the gerbils were fed diets containing different concentrations of doxycycline (range, 0 to 175 mg/kg doxycycline in chow). In subsequent experiments, animals were fed a diet containing 25 mg/kg doxycycline or drug-free chow for specific time periods.

### Processing of gastric tissue.

At the end of the experiments, gerbil stomachs were excised and processed to retain glandular portions of the stomach (corpus and antrum), and the nonglandular portion of stomach (forestomach) was discarded. The glandular section of stomach was then cut open along the lesser curvature, and three longitudinal strips from each stomach were processed for H. pylori culture, analysis of gene expression, and histologic analysis as described below.

### Bacterial colonization density.

Longitudinal strips of stomach were homogenized with a tissue tearor (Biospec Products, Inc.) in sulfite-free brucella broth supplemented with 10% fetal bovine serum (FBS) until tissues were completely homogenized. H. pylori colonization density (CFU/gram of stomach tissue) was determined by plating serial dilutions of the tissue homogenates on TSA plates supplemented with 5% sheep blood, 50 μg/ml vancomycin, 100 μg/ml bacitracin, 10 μg/ml nalidixic acid, and 2 μg/ml amphotericin and culturing in microaerobic conditions (79). At least five single colonies of H. pylori output strains from each animal were pooled together and frozen for subsequent analyses.

### Histology.

Longitudinal strips of stomach tissue were fixed in 10% formalin overnight, embedded in paraffin, sectioned, and stained with hematoxylin and eosin. Histologic sections of gastric tissues were analyzed by a gastrointestinal pathologist in a blinded fashion. The tissue sections were evaluated for gastric inflammation (gastritis), presence of lymphoid follicles, gastric ulceration, dysplasia, and gastric adenocarcinoma in corpus and antrum (80). Histologic scoring data for gerbils experimentally infected with H. pylori are presented only for the gerbils that were successfully colonized, based on culture and/or use of a modified Steiner stain. Histological scores (0, 1, 2, and 3 each representing absent, mild, moderate, and marked inflammation, respectively) were assigned to evaluate acute (neutrophils) and chronic (mononuclear leukocytes) inflammation in both the corpus and antrum, and these scores were added together to yield a cumulative score of 0 to 12 (20, 21, 79–81).

### Analysis of *cagUT* expression.

Expression of *cagUT* and control genes was analyzed in H. pylori cultured *in vitro*, as well as in gastric tissue from H. pylori-infected gerbils, as described in Text S1 and Table S1 in the supplemental material.

10.1128/mBio.01296-20.9 TABLE S1 Oligonucleotide sequences used for qRT-PCR. Download Table S1, DOCX file, 0.02 MB. Copyright © 2020 Lin et al. 2020 Lin et al. This content is distributed under the terms of the Creative Commons Attribution 4.0 International license.

10.1128/mBio.01296-20.10 TABLE S2 Frequency of most severe diagnosis in uninfected and infected animals receiving various concentrations of doxycycline. Download Table S2, DOCX file, 0.02 MB. Copyright © 2020 Lin et al. 2020 Lin et al. This content is distributed under the terms of the Creative Commons Attribution 4.0 International license.

### Western blot analysis.

H. pylori strains were cultured with and without ATc or with various concentrations of doxycycline (0 to 40 ng/ml). H. pylori lysates were separated by sodium dodecyl sulfate-polyacrylamide gel electrophoresis (SDS-PAGE), and the proteins were transferred to a nitrocellulose membrane. Subsequently, the membrane was immunoblotted with a rabbit polyclonal anti-CagT antiserum and anti-Hsp60 antiserum (82), and developed using goat anti-rabbit IgG labeled with horseradish peroxidase (HRP) and enhanced chemiluminescence.

### NF-κB activation.

NF-κB activation was analyzed using AGS cells stably expressing a luciferase-based NF-κB reporter (83). Briefly, the reporter cell line was cocultured with H. pylori strains (multiplicity of infection [MOI] of 100:1) for 2 to 3 h at 37°C. H. pylori strains were cultured in the presence or absence of ATc for 24 to 48 h prior to testing NF-κB activation. Luminescence was measured using the Steady-Glo kit (Promega) on a BioTek FLx800 plate reader.

### CagA translocation assay.

CagA translocation into AGS gastric epithelial cells was analyzed using previously described methods (84–90). Briefly, H. pylori strains were cocultured with AGS cells at a MOI of 100:1 for 4 to 6 h at 37°C. CagA translocation was detected using an anti-phosphotyrosine antibody (anti-PY99; Santa Cruz Biotechnology) for tyrosine phosphorylation of CagA, and CagA was detected using anti-CagA antibody (Santa Cruz Biotechnology).

### Statistical analyses.

GraphPad Prism and R were used to perform the statistical analyses. A Mann-Whitney test was used to assess differences among groups in inflammation (two groups) or NF-κB activation. The Kruskal-Wallis test with Dunn’s multiple-comparison test was used to assess differences among groups in inflammation (multiple groups) and bacterial burden (multiple groups). Fisher’s exact test with Benjamini-Hochberg multitest correction was used to analyze differences among groups in incidence of gastric diseases. An unpaired *t* test with Welch’s correction was used to evaluate numbers of lymphoid follicles/aggregates.

Bayesian z-scores and a standard z-test (multiple test correction using the Benjamini-Hochberg method) were used to evaluate gene expression RT-PCR data. Specifically, quantitative RT-PCR data were analyzed using generalized linear mixed models based on log-normal Poisson error distribution and fitted using Markov chain Monte Carlo analysis (91). Data were fit using informed models and data from control genes. Amplification efficiencies were determined based on analysis of amplification of targets over a 6-log range of template concentrations using purified H. pylori genomic DNA and optimized primer annealing temperatures. The credible intervals were determined by the MCMC.qpcr package in R. Based on the Bayesian framework, a credible interval (or credible limit) is an analog of a confidence interval in frequentist statistics. The 95% credible limit indicates there is a 95% probability of the true value falling with this parameter.

### Ethics statement.

All animal experiments were approved by the Vanderbilt University Institutional Animal Care and Use Committee (protocol M1700055-00).
