# Supplementary material for: Temporal Control of the Helicobacter pylori Cag Type IV Secretion System in a Mongolian Gerbil Model of Gastric Carcinogenesis
Source: mBio. 2020 Jun 30;11(3):e01296-20. doi: 10.1128/mBio.01296-20 (PMC7327173; doi:10.1128/mBio.01296-20)
Supplement: TEXT S1 [file mBio.01296-20-s0001.docx]

**Supplemental Methods**

**Generation of *H. pylori* strains in which Cag T4SS** **activity can be conditionally regulated**

In a previous study, we reported methods for conditional expression of the *cagUT* operon and Cag T4SS activity in *H. pylori* 26695 based on use of the TetR/*tetO* system ([2](#_ENREF_2)). In the current study, we introduced similar TetR/*tetO* elements into the gerbil-adapted *H. pylori* strain 7.13 ([1](#_ENREF_1)). The codon-optimized *tetR* and a chloramphenicol resistance determinant were cloned into the intergenic region between *mdaB* and *hydA* derived from strain G27 (corresponding to HP0630 and HP0631 in strain 26695) to generate plasmid pMM685 (Table 1). Strain 7.13 was then transformed with plasmid pMM685 and chloramphenicol-resistant colonies were selected, resulting in *H. pylori* strain VM127. PCR and DNA sequencing confirmed that homologous recombination via double crossover resulted in insertion of *tetR* into the appropriate site in the *H. pylori* chromosome. Strain VM127 was then used to infect gerbils to ensure that the strain retained its capacity for gastric colonization.

A gerbil output strain derived from VM127, designated VM127-Mu, was modified by transforming this strain with plasmid pMM680, which contains three copies of *tetO* introduced adjacent to the *cagUT* promoter ([2](#_ENREF_2)). Kanamycin-resistant colonies were selected, and PCR and DNA sequencing were used to confirm the introduction of the recombinant *cagU* promoter. Multiple colonies of the resulting strains, designated VM197-201, were used to infect gerbils. Pools of *H. pylori* colonies cultured from two infected gerbils were designated VM202-203 (Suppl. Fig. S1). VM202-203 was then used for experimental infection of gerbils.

**Analysis of *cagUT* expression**

For analysis of gene expression in *H. pylori* cultured *in vitro,* strains were cultured for 16 hours in sulfite-free Brucella Broth supplemented with cholesterol. RNA was isolated using Trizol (Life Technologies) according to the manufacturer’s protocol. Purified RNA (0.25 μg) was treated with Turbo DNA-free (Life Technologies). One half of each sample was used for first strand cDNA synthesis using Superscript III (Life Technologies) with random hexamer priming followed by RNase H treatment. The other half of each sample was treated in the same manner, except that reverse transcriptase was omitted. Quantitative real-time PCR (qRT-PCR) was performed using iQ SYBR Green Supermix (Bio-Rad) and oligonucleotide primers for *cagU* and control genes *lnt*, *lpxD*, and *prfA* (Suppl. Table S1). Control genes were chosen based on analysis of RNA-seq data, indicating stable expression of selected genes in response to *in vitro* stress (high salt conditions) and transcript abundance that was comparable (*lpxD* and *prfA*) to *cagU*, or approximately 0.1x as abundant (*lnt*) compared with *cagU*.

For analysis of gene expression from infected animal tissue, Trizol (Life Technologies) was added to stomach tissue along with 1 mm zirconium oxide (zirconia) beads (BioSpec Products). Samples were homogenized by vortexing for 5 minutes and then frozen at -70^o^C. RNA was extracted from the homogenized samples using the manufacturer’s instructions. Purified RNA (40 μg) was treated with Turbo DNA-free (Life Technologies). One half of each sample (approximately 20 μg) was used for first strand cDNA synthesis using Superscript III (Life Technologies) with random hexamer priming followed by RNase H treatment. Duplicate samples (approximately 20 μg) were treated in the same manner, except that reverse transcriptase was omitted. Nucleic acids were then recovered in 60 μl water using AxyPrep Mag PCR Clean-up (Axygen). The recovered nucleic acids were then divided into 3 aliquots: 6 μl was used for qRT-PCR using gerbil GAPDH-specific primers (as one indicator of sample quality); 27 μl was used for 12 cycles of PCR using outer primers for nested PCR of *H. pylori lpxD*; and 27 μl was used for 12 cycles of PCR using outer primers for nested PCR of *H. pylori cagU* (Suppl. Table S1). The *H. pylori* outer PCR reaction products were purified using AxyPrep Mag PCR Clean-up (Axygen). The purified outer PCR products were then used for qRT-PCR of *lpxD* or *cagU* using inner primers (Suppl. Table S1), in triplicate. Quantitative RT-PCR was performed using iQ SYBR Green Supermix (Bio-Rad).

1. **Franco, A. T., D. A. Israel, M. K. Washington, U. Krishna, J. G. Fox, A. B. Rogers, A. S. Neish, L. Collier-Hyams, G. I. Perez-Perez, M. Hatakeyama, R. Whitehead, K. Gaus, D. P. O'Brien, J. Romero-Gallo, and R. M. Peek.** 2005. Activation of β-catenin by carcinogenic *Helicobacter pylori*. Proceedings of the National Academy of Sciences of the United States of America **102:**10646-10651.

2. **McClain, M. S., S. S. Duncan, J. A. Gaddy, and T. L. Cover.** 2013. Control of gene expression in *Helicobacter pylori* using the Tet repressor. Journal of microbiological methods **95:**336-341.

**Supplemental Figure Legends**

**Supplemental Figure S1**: Introduction of *tetR* and *tetO* in the engineered strain VM202-203. The codon-optimized *tetR* was introduced into the intergenic region between *mdaB* and *hydA* derived from strain G27. Three copies of *tetO* were introduced upstream of the *cagUT* operon to regulate *cagUT* gene expression. The DNA sequence of *tetO* in the modified promoter region of *cagUT* is described in detail in ([2](#_ENREF_2)).

**Supplemental Figure S2**: Pilot experiment analyzing transcript abundance of *cagU* and a control gene (*lpxD)* in stomach tissues of infected gerbils receiving chow containing 50 mg/kg doxycycline (n=2) compared to infected gerbils receiving chow containing 0 mg/kg doxycycline (n=3). Values represent the mean (and 95% credible limit) log_2_ fold change. Bayesian z-scores were calculated, and a standard z-test performed to derive two-tailed p-values. The p-values (0.922 and 0.054 for *lpxD* and *cagU*, respectively) were corrected for multiple testing using the Benjamini-Hochberg method.

**Supplemental Figure S3**: Stability of the Cag T4SS system *in vivo*. Gerbils were infected with *H. pylori* VM202-203 and fed diets containing a range of doxycycline concentrations, as described in Figure 2. (A) *H. pylori* strains cultured from infected animals fed a normal (drug-free) diet for 3 months were tested for capacity to stimulate NF-κB activation in AGS reporter cells. The label 1-0D indicates animal number 1 fed chow containing 0 mg/kg doxycycline. (B) NF-κB activation induced by output strains cultured from infected animals fed a diet containing 10 mg/kg or 25 mg/kg doxycycline for 3 months. The labels 1-10D and 1-25D indicate strains cultured from animals fed chow containing 10 mg/kg or 25 mg/kg doxycycline, respectively. Strain 7.13 was used as a positive control and VM196 as a negative control. The individual data represent results of two or three independent experiments with multiple technical replicates. Values represent means ± standard error of the mean (SEM). Significance was determined using Mann Whitney test for *A, B.** p≤0.05, ** p≤0.01, *** p≤0.001, and **** p≤0.0001.

**Supplemental Figure S4**: Gastric inflammation in antrum and corpus of infected gerbils receiving diets containing the indicated concentrations of doxycycline. Gerbils were infected with *H. pylori* VM202-203 and fed diets containing a range of doxycycline concentrations, as described in Figure 2. (A, B) Acute and chronic inflammation in the antrum. (C, D) Acute and chronic inflammation in the corpus. (E) Lymphoid follicles/aggregates in the glandular portion of stomach. Each symbol represents results for an individual animal*.* Mann Whitney test for *A, B, C, D* or unpaired t test with Welch’s correction for *E* were used to calculate significance. ** p<0.01; *** p<0.001.

**Supplemental Figure S5**: Stability of the TetR/*tetO* system *in vivo*. Gerbils were infected with *H. pylori* VM202-203 and fed various diets, as described in Figure 3. (A) *H. pylori* strains cultured from infected animals fed a drug-free diet for 3 months were tested for their capacity to stimulate NF-κB activation in AGS cells. (B) NF-κB activation induced by output strains cultured from infected animals fed a diet containing 25 mg/kg doxycycline for 3 months. Strain 7.13 was used as a positive control and strain VM196 as a negative control. In parallel, the output strains from individual animals were grown in the absence or presence of ATc for 24-48 hours prior to testing NF-κB activation. The data represent results of two or three independent experiments with multiple technical replicates. Values represent mean ± standard errors of the mean (SEM). Significance was determined using Mann Whitney test*.* * p≤0.05, ** p≤0.01, *** p≤0.001, and **** p≤0.0001.

**Supplemental Figure S6**: Acute and chronic inflammation in response to Cag T4SS activity during specific stages of infection. Gerbils were infected with *H. pylori* VM202-203 and fed various diets, as described in Figure 4. (A, B) Acute and chronic inflammation in the antrum. (C, D) Acute and chronic inflammation in the corpus. (E) Lymphoid follicles/aggregates in the glandular portion of the stomach. Each symbol represents results for an individual animal*.* Kruskal-Wallis test with Dunn’s multiple-comparison test was used to calculate significance for *A, B, C* and *D*. * p<0.05; ** p<0.01; *** p<0.001.

**Supplemental Figure S7**: Gastric inflammation in infected gerbils receiving chow containing 0 or 25 mg/kg doxycycline during defined stages of infection. Gerbils were infected with *H. pylori* VM202-203 and fed various diets, as described in Figure 4. (A-D) *H. pylori*-infected animals or uninfected animals received the indicated diets throughout the 3-month time period. (E) *H. pylori*-infected animals received drug-free chow during the first 3 weeks of infection, followed by chow containing doxycycline for the subsequent 10 weeks. (F) *H. pylori*-infected animals received chow containing doxycycline for the first 3 weeks of infection followed by drug-free chow for the subsequent 10 weeks. The panels depict gastric mucosa from the antrum, showing normal histology in uninfected gerbils receiving 0 mg/kg doxycycline (A), uninfected gerbils receiving 25 mg/kg doxycycline (B), infected gerbils receiving 0 mg/kg doxycycline (C); severe gastric inflammation and dysplastic glands in infected gerbils receiving 25 mg/kg doxycycline (D); and severe inflammation and lymphoid follicles in infected gerbils receiving doxycycline for the indicated time periods (E, F). Magnification, 100X.
