# Supplementary figures and images for: Temporal Control of the Helicobacter pylori Cag Type IV Secretion System in a Mongolian Gerbil Model of Gastric Carcinogenesis
Source: mBio. 2020 Jun 30;11(3):e01296-20. doi: 10.1128/mBio.01296-20 (PMC7327173; doi:10.1128/mBio.01296-20)

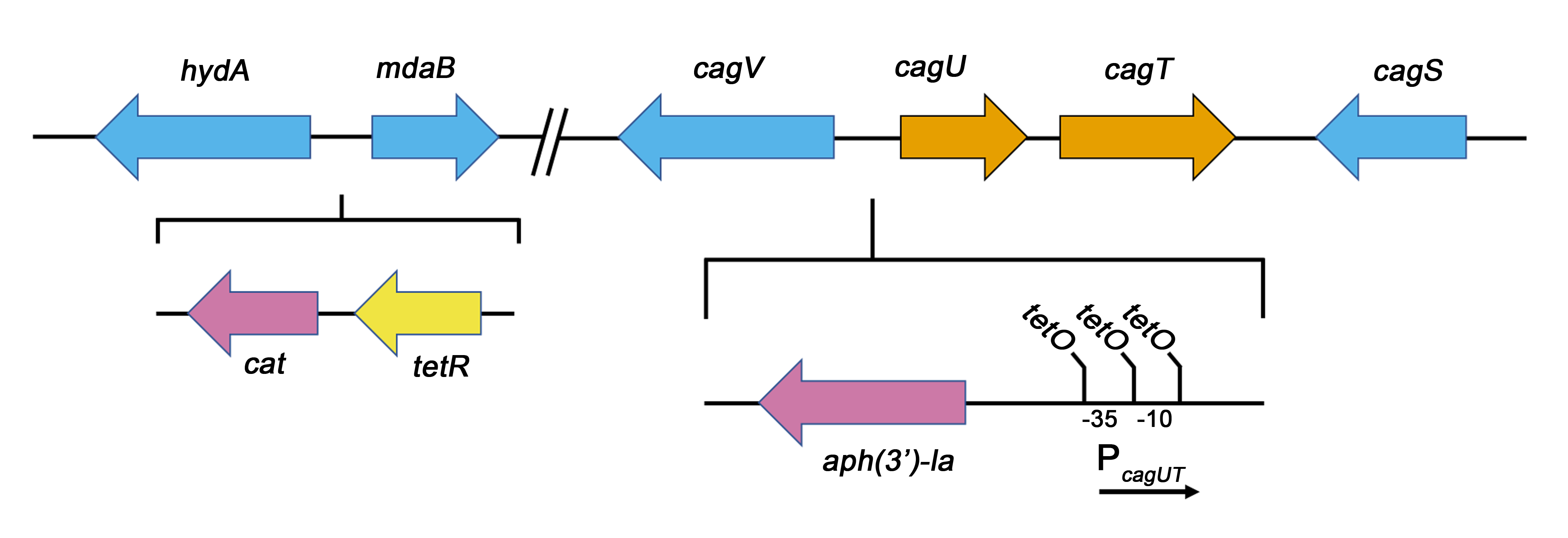

Supplement: FIG S1 [file mBio.01296-20-sf001.tif]

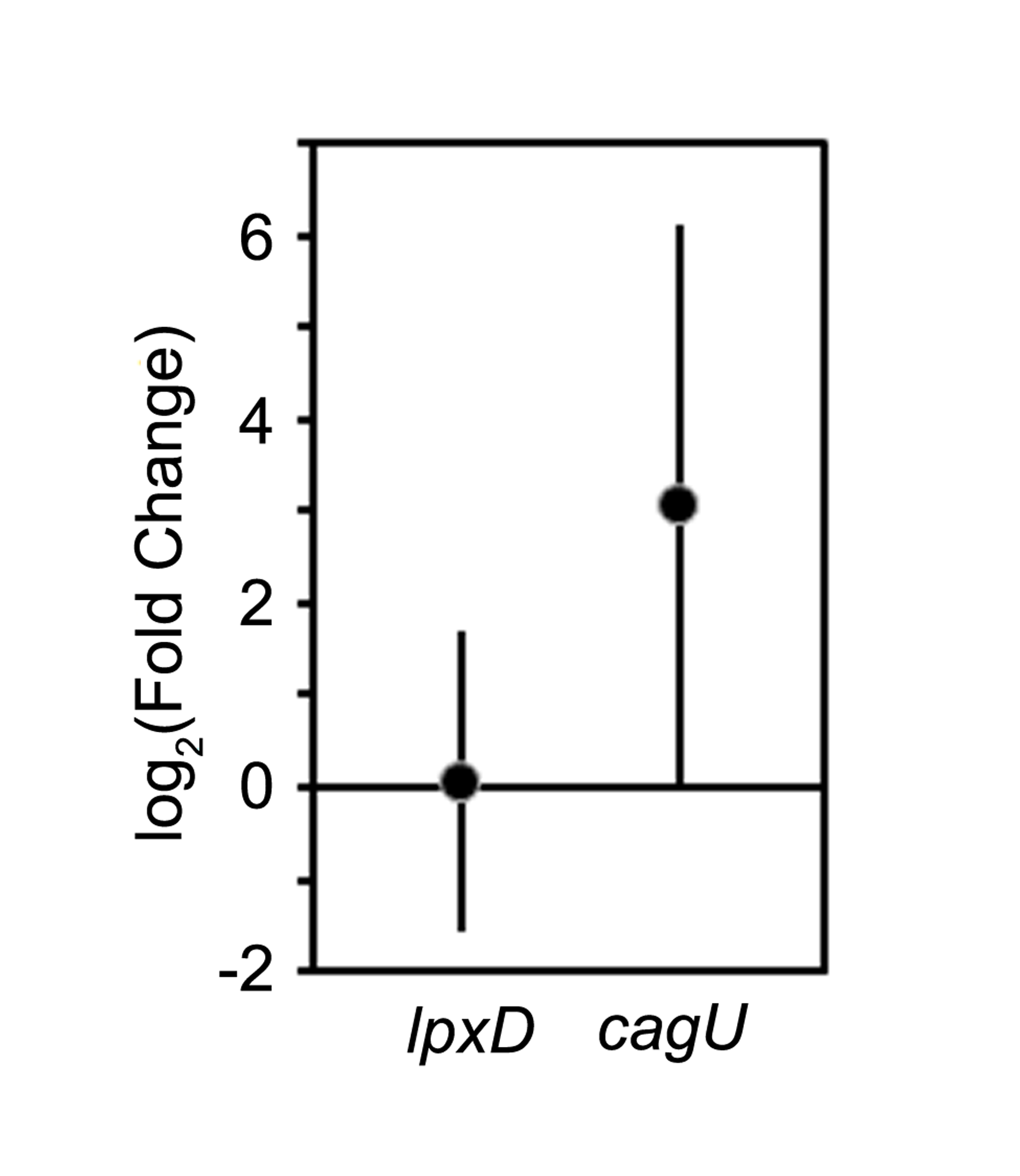

Supplement: FIG S2 [file mBio.01296-20-sf002.tif]

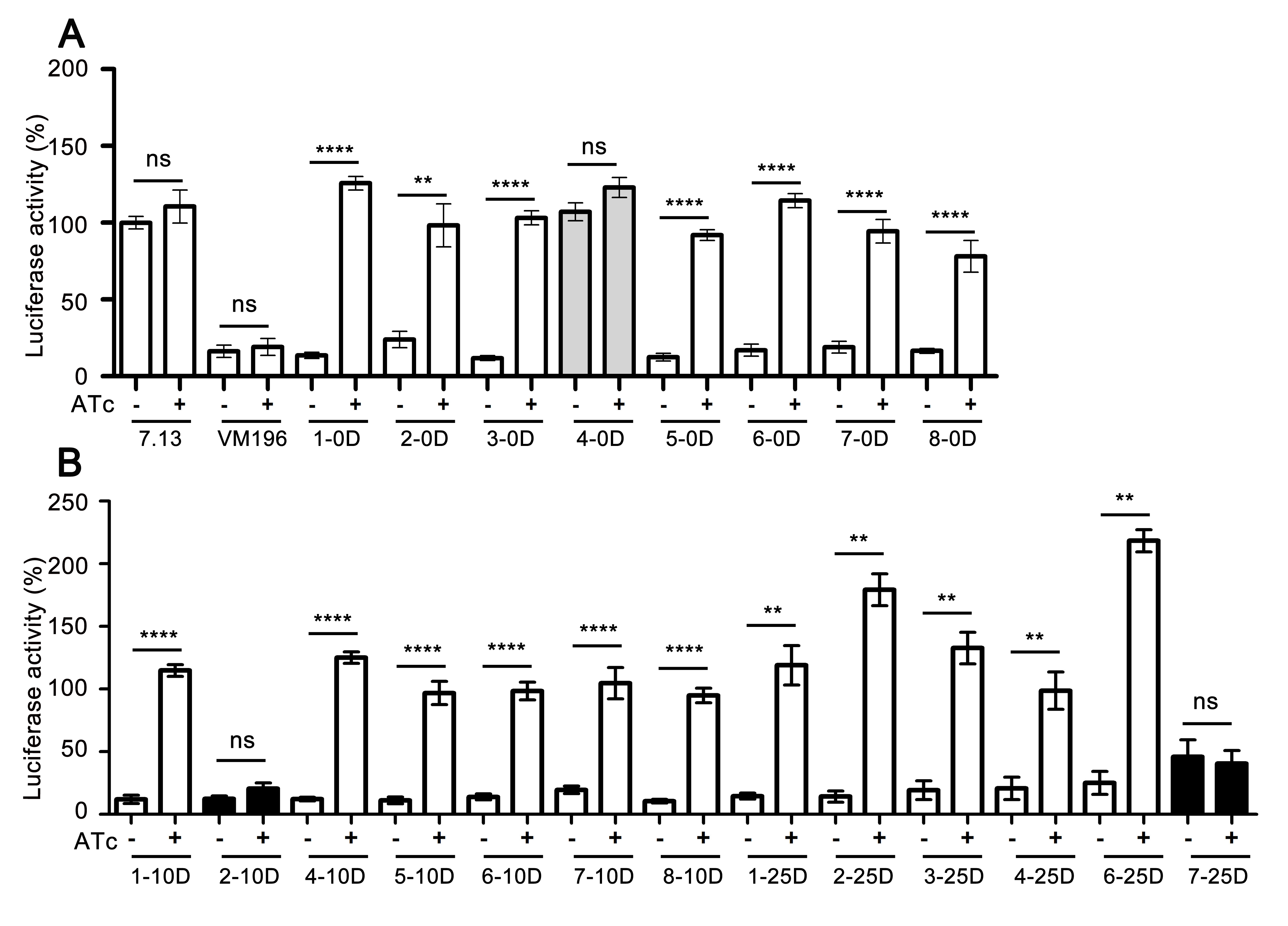

Supplement: FIG S3 [file mBio.01296-20-sf003.tif]

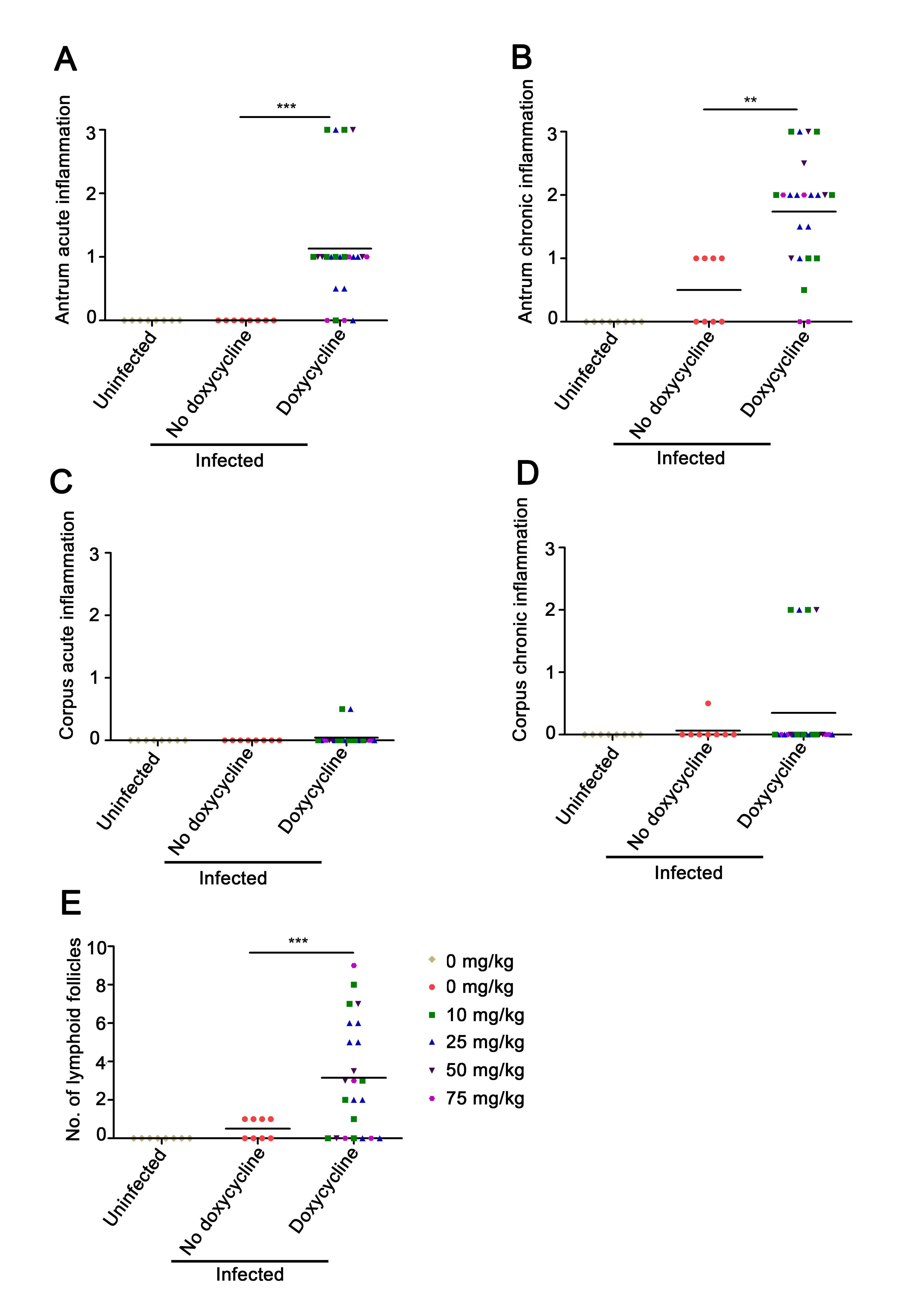

Supplement: FIG S4 [file mBio.01296-20-sf004.tif]

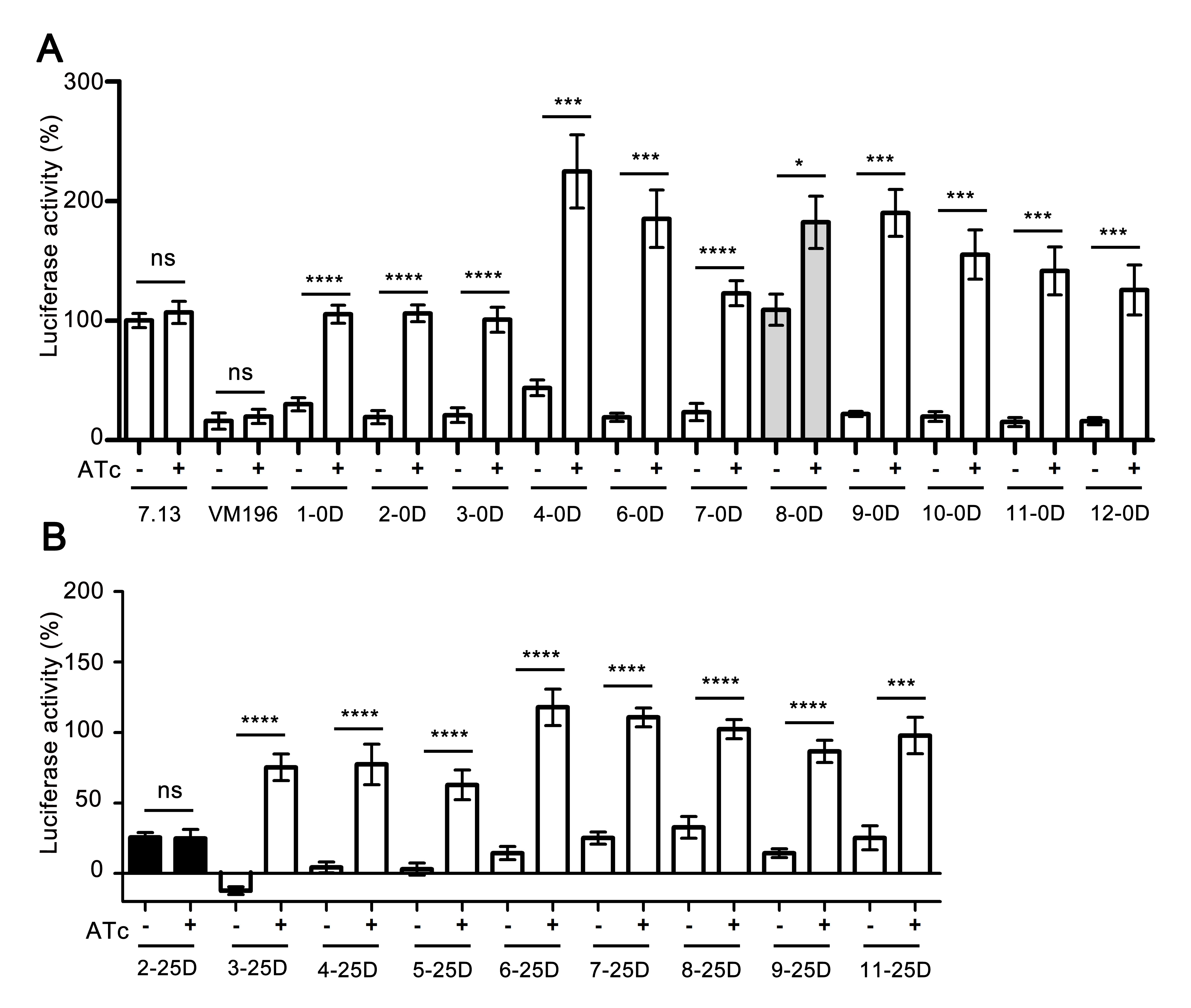

Supplement: FIG S5 [file mBio.01296-20-sf005.tif]

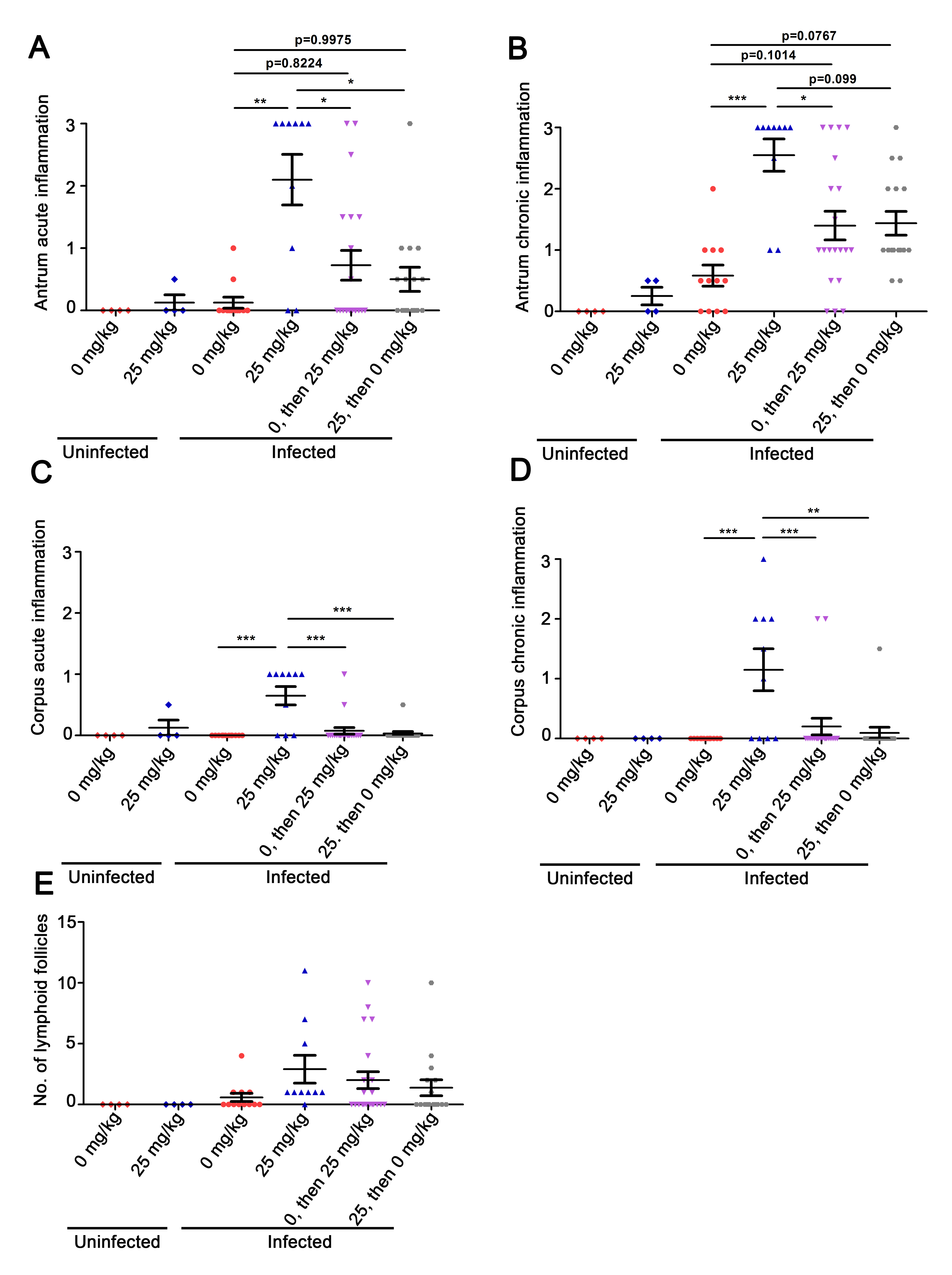

Supplement: FIG S6 [file mBio.01296-20-sf006.tif]

## Slide 1
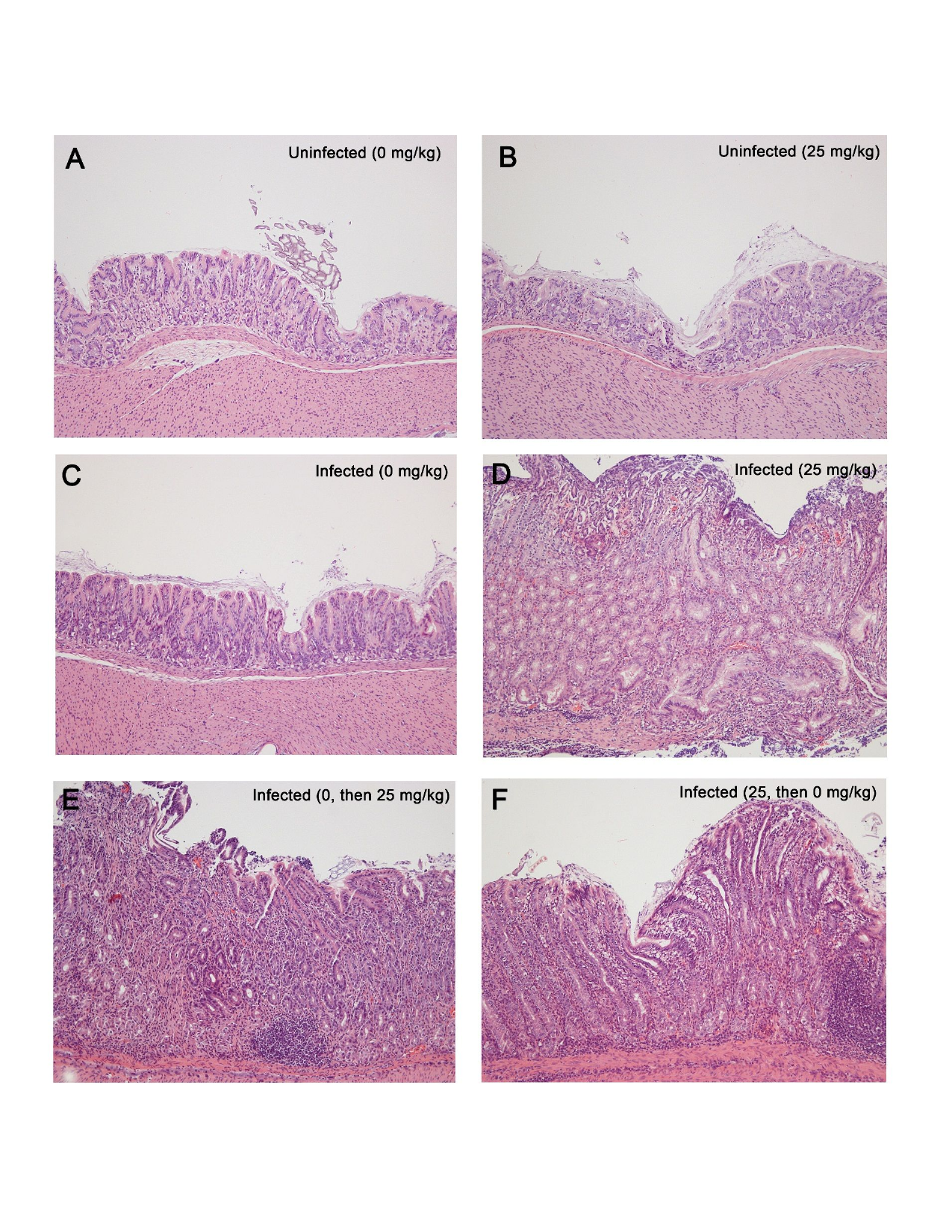

Supplement: FIG S7 [file mBio.01296-20-sf007.ppt]
