# Supplementary material for: Temporal Control of the Helicobacter pylori Cag Type IV Secretion System in a Mongolian Gerbil Model of Gastric Carcinogenesis
Source: mBio. 2020 Jun 30;11(3):e01296-20. doi: 10.1128/mBio.01296-20 (PMC7327173; doi:10.1128/mBio.01296-20)
Supplement: TABLE S1 [file mBio.01296-20-st001.docx]

Supplemental Table S1. Oligonucleotide sequences used for qRT-PCR

| Species | Gene | Forward | Reverse |
| --- | --- | --- | --- |
| *Meriones unguiculatus* | Gapdh | CATGGCCTTCCGAGTTCCT | TTCTGCAGTCGGCATGTCA |
| *H. pylori* | *lnt* | cttccaaacccgcttattca | agttgaaaagttcgcgctgt |
| *H. pylori* | *prfA* | GCGATGAAGCGGGCATTTTT | GAGCCTTGAATACACGCCCT |
| *H. pylori* | *lpxD* Outer | TGACCAAGCGCGTTACCTTA | GTGCGCATAACCAAAGCCAT |
| *H. pylori* | *lpxD* Inner | AAGTGCCAAAACGCATGCAA | TTCGCCAATTTCTACGCCCT |
| *H. pylori* | *cagU* Outer | TGGACCTAATCCGCTAAACG | TTCTTTGCTTGGCTTGTCCT |
| *H. pylori* | *cagU* Inner | GGGAGTTTAGGGGAGCAAAGA | TCTGCATCAGAAGTTCATACCTT |
