# Supplementary material for: Temporal Control of the Helicobacter pylori Cag Type IV Secretion System in a Mongolian Gerbil Model of Gastric Carcinogenesis
Source: mBio. 2020 Jun 30;11(3):e01296-20. doi: 10.1128/mBio.01296-20 (PMC7327173; doi:10.1128/mBio.01296-20)
Supplement: TABLE S2 [file mBio.01296-20-st002.docx]

Supplemental Table S2: Frequency of most severe diagnosis in uninfected and infected animals receiving various concentrations of doxycycline

| Animal group^a^ | Normal histology | Gastritis | Dysplasia | Cancer | No. of successfully colonized animals^b^ | Total no. of animals used |
| --- | --- | --- | --- | --- | --- | --- |
| Uninfected | 8 | 0 | 0 | 0 | 0 | 8 |
| Infected (0 mg/kg) | 4 | 4 | 0 | 0 | 8 | 8 |
| Infected (10 mg/kg) | 1 | 4 | 2 | 1 | 7 | 8 |
| Infected (25 mg/kg) | 0 | 7 | 0 | 1 | 8 | 8 |
| Infected (50 mg/kg) | 1 | 6 | 0 | 1 | 4 | 8 |
| Infected (75 mg/kg) | 4 | 3 | 0 | 0 | 4 | 7 |

^______________________________________________________________________________________________________________________________________^

^a^ Animals were infected with *H. pylori* VM202-203 and fed diets containing the indicated doxycycline concentrations. Uninfected animals in this experiment received drug-free chow.

^b^*H. pylori* colonization was evaluated by culturing *H. pylori* and/or Steiner stain.
